# Ultrasound radiomics for preoperative evaluation of Ki-67 proliferation index in papillary thyroid carcinoma

**DOI:** 10.3389/fonc.2026.1881562

**Published:** 2026-09-03

**Authors:** Hui Shi, Jiayi Qiu, YuLu Wu, Ying Zhang, HengQi Zhang, YunYun Liu, YiTong Li, JiaHui Ni, ChongKe Zhao, HuiXiong Xu, LiPing Sun, LeHang Guo, YiFeng Zhang

**Affiliations:** 1 Department of Medical Ultrasound, Shanghai Tenth People's Hospital, School of Medicine, Tongji University, Shanghai, China; 2 Department of Ultrasound, Zhongshan Hospital, Fudan University, Shanghai, China; 3 Department of Ultrasound, Shanghai General Hospital, Shanghai Jiao Tong University, Shanghai, China

**Keywords:** BRAF and TERT dual mutations, high Ki-67 expression, papillary thyroid carcinoma, radiomics, ultrasound features

## Abstract

**Purpose:**

To explore the predictive value of radiomics features, ultrasound (US), and gene mutation status based on interpretable random forest (RF) models for predicting high Ki-67 expression in papillary thyroid carcinoma (PTC).

**Methods:**

This retrospective analysis included 627 patients with surgically confirmed PTC who underwent testing for BRAF V600E and TERT promoter mutations, as well as immunohistochemical assessment of Ki-67 expression from December 2015 to June 2023. The eligible patients were randomly divided into a training set and a testing set at a ratio of 7:3 according to their binary Ki-67 expression status. A random forest model was constructed using both individual and combined ultrasound radiomics features, conventional ultrasound features, and genetic mutation status to predict high Ki-67 expression in PTC. Using AUC, Brier score and decision curve analysis to verify the clinical utility of the model.

**Result:**

The Rad+US+Gene model demonstrated superior predictive accuracy for high Ki-67 expression, achieving the highest accuracy and AUC, along with the lowest Brier score. In the testing cohort, the Rad+US+Gene model attained an accuracy of 0.883, outperforming Rad+US, US and Rad (0.851). Its AUC reached 0.904, markedly exceeding those of Rad+US (0.854), US (0.823), and Rad (0.851). Regarding calibration, the Rad+US+Gene model also yielded the lowest Brier score (0.0822), compared with Rad+US (0.1073), US (0.1061), and Rad (0.1232), indicating superior predictive accuracy and stability.

**Conclusion:**

The integrated model combining radiomics, US features and genetic mutations achieves favorable predictive performance, presenting a new method for the preoperative assessment of Ki-67 expression level in PTC.

## 1 Introduction

Papillary thyroid carcinoma (PTC) accounts for the majority of thyroid malignancies (1). Clinically, approximately 90% of patients with PTC fall into the low-risk category (2), whereas a minority experience aggressive tumor progression, recurrence, and distant metastasis. This marked prognostic heterogeneity underscores the critical need for precise risk stratification and individualized management and treatment for PTC patients.

Biomarkers in oncology are broadly classified as prognostic or predictive, each offering distinct clinical utility. Prognostic biomarkers, such as BRAF V600E and TERT promoter mutations, has corroborated closely associated with aggressive clinicopathological features (3–5) and poor prognosis (6, 7) of PTC. In contrast, predictive biomarkers offer dynamic phenotypic and functional readouts that complement static genotyping and inform preoperative decisions. However, mutations alone cannot fully mirror PTC biology, as they drive oncogenesis indirectly via downstream protein and phenotypic modulation (8). Therefore, it is necessary to predict the phenotypic biomarkers before PTC surgery.

Ki-67, a core nuclear antigen and direct indicator of tumor proliferation, is a key phenotypic consequence of BRAF V600E and TERT promoter mutations. Previous studies have suggested that these mutations upregulate Ki-67 expression by regulating cell proliferation and differentiation pathways (9, 10), and elevated >5% Ki-67 levels are closely associated with tumor aggressiveness and prognosis (11–13). However, the precise relationship and regulatory mechanisms between these mutations and Ki-67 remain unclear. Current preoperative PTC assessment focuses on genetics (14, 15), without incorporating phenotypic biomarkers, thereby limiting its predictive accuracy. Thus, elucidating the mutation-Ki-67 relationship and establishing a reliable preoperative assessment method are essential to guide individualized therapeutic decision-making.

Current Ki-67 evaluation depends on postoperative pathology or preoperative fine-needle aspiration (FNA) or core-needle biopsy (CNB) immunohistochemistry. However, FNA and CNB are particularly prone to sampling heterogeneity (16), which limits their ability to reflect global proliferative (17) and precludes longitudinal monitoring (18). Ultrasound (US), the first-line imaging modality for PTC, is noninvasive, reproducible, and widely accessible (19). Although current guidelines enable preliminary risk stratification through visual US assessment, though visual evaluation alone cannot reliably identify microstructural, genetic, or protein-level alterations. But recently, ultrasound radiomics has emerged as a promising approach for high-throughput extraction of quantitative texture, morphological, and grayscale features. Combined with machine learning, this technique converts ultrasound images into high-dimensional data (20) capable of noninvasively reflecting microscopic biological information, including tumor genetics mutations (21, 22), recurrence and lymph node metastasis (23, 24) in PTC. While preoperative Ki-67 expression has been achieved in breast cancer (25), liver cancer (26), and other tumors (27), analogous research in PTC, especially regarding the association between BRAF, TERT mutation status and Ki-67 expression, has been limited.

Therefore, this study aims to develop a machine learning model combined radiomics and conventional ultrasound features for noninvasive preoperative prediction of Ki-67 expression associated with BRAF V600E and TERT promoter mutations in PTC. The models were constructed to investigate the correlation among imaging phenotypes, molecular mutations, and protein alterations, thereby enabling improved preoperative risk stratification, molecular and protein assessment, and personalized treatment planning.

## 2 Materials and methods

### 2.1 Patients and study population

This retrospective study was approved by the Ethics Committee of Shanghai Tenth People’s Hospital (No.2022XJS36). All procedures were performed in accordance with the Declaration of Helsinki. Written informed consent was waived because of the retrospective design and anonymized patient data.

**Figure 1:**
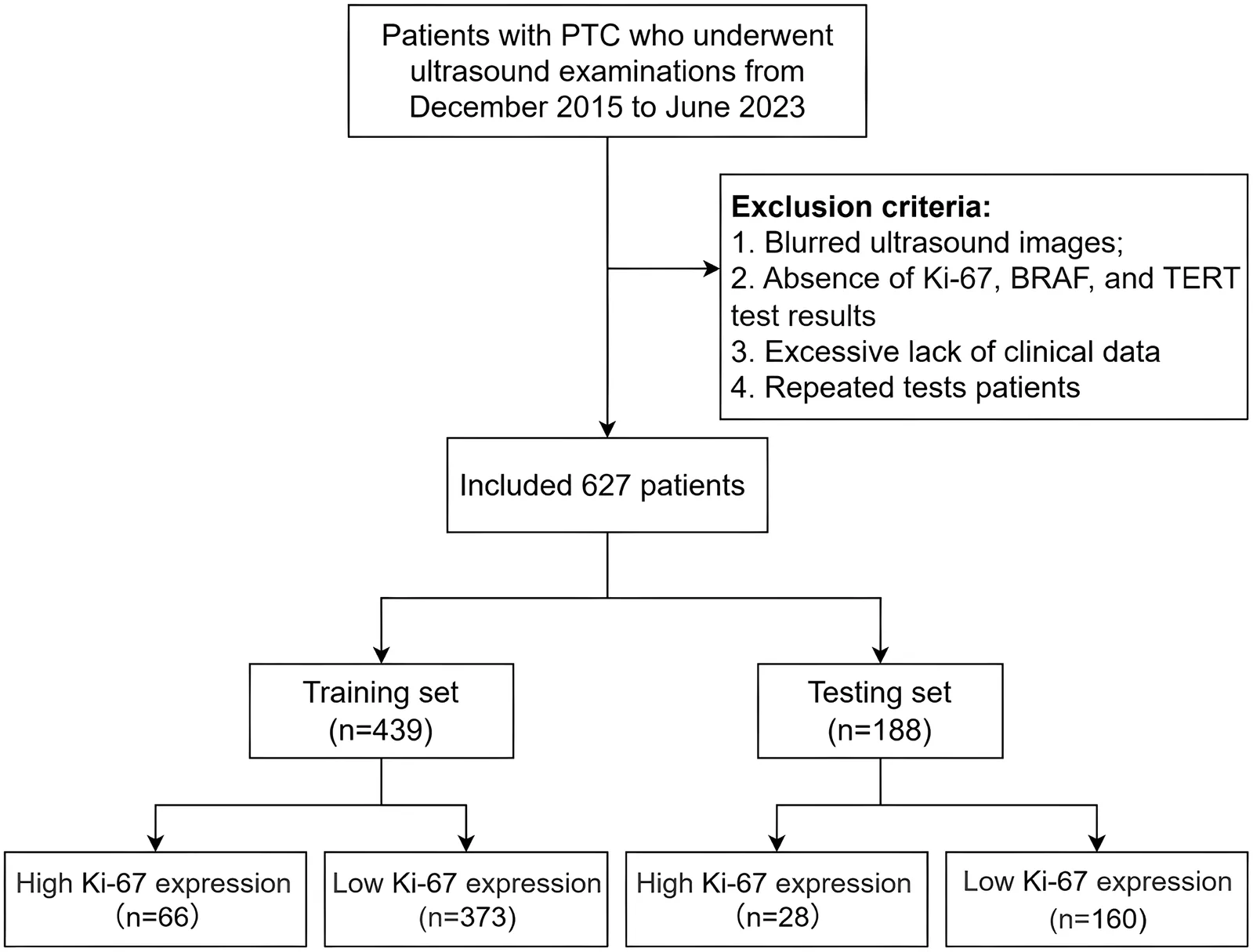
Flowchart of patient enrollment and grouping.

This study retrospectively included patients with thyroid nodules who underwent thyroidectomy from December 2015 to June 2023. Inclusion criteria included (1): Diagnosed with PTC by postoperative pathology, and postoperative immunohistochemical testing including Ki-67 proliferation level (2); Complete routine ultrasound examination within one month before surgery, with complete and clear image data (unobstructed, and identifiable lesion boundaries) (3); BRAF and TERT gene testing has been performed through FNA or histopathology (4); Complete clinical data, including gender, age, tumor size, capsule infiltration, lymph node metastasis, etc (5); There is no history of thyroidectomy, ablation, or chemotherapy before image acquisition. Exclusion criteria were: (1) blurry ultrasound images, inability to segment lesions, and missing blood flow signals; (2) Unclear pathological diagnosis, missing or invalid Ki-67 test results; (3) There are too many missing clinical data and gene mutation test results to complete statistical analysis; (4) For patients who have been readmitted or tested repeatedly, the Ki-67 detection results corresponding to the tissue pathology after the first PTC surgery will be used. Finally, 627 patients were included (441 females and 186 males; average age 45.1 ± 13.8 years). All patients were stratified by binary Ki-67 expression status and randomly assigned to training and testing sets at a 7:3 ratio, ensuring that the original proportions of high (15.0%) and low (85.0%) expression were preserved in both cohorts. (**Figure 1**).

### 2.2 Ultrasound image acquisition

Grayscale and color Doppler images distribution of different high-resolution ultrasound scanners (French Supersonic, GE Logiq E9) were shown in **Supplementary Table 1**. For each nodule, cross-sectional, longitudinal, and flow images were obtained, covering the entire nodule and its surrounding tissue. In order to obtain the best imaging quality of the target lesion, and reduce the interference caused by poor image quality, all ultrasonographers involved in image acquisition underwent standardized training in thyroid imaging. Ultrasound images were acquired with real-time optimization of scanning parameters based on individual patient anatomy and lesion characteristics. The adjustable parameters included probe frequency, imaging depth, gain, focal position, and time gain compensation. These adjustments ensured optimal visualization of key nodule features (margin, shape, echogenicity, calcifications, and vascularity, etc.), thereby maintaining image integrity and diagnostic suitability for subsequent analysis.

### 2.3 Data collection and visual assessment

A set of clinical and ultrasound features was collected. Clinical variables included age, sex, Ki-67 expression, multifocality, lymph node metastasis, and capsule infiltration (all recorded as binary: yes or no). Ultrasound features covered tumor location (upper, middle, or other), size, multifocality (yes or no), taller-than-wide (yes/no), margin (irregular or regular), capsule infiltration (yes or no), composition (solid or mixed solid), echogenicity (markedly hypoechoic or not), calcification (none, microcalcifications, or macrocalcifications), posterior acoustic features (none, enhancement, or attenuation), blood flow signal (rich or poor), and vascular distribution (peripheral, central, or mixed).

### 2.4 Radiomics features extraction and screening

Ultrasound images were preprocessed to ensure standardization. In adherence to the Image Biomarker Standardization Initiative (IBSI) guidelines, all preprocessing and feature extraction steps were designed to be IBSI-compliant where applicable. Image preprocessing did not apply forced isotropic resampling to preserve original acquisition resolution; instead, all images were spatially aligned to a common coordinate system via rigid registration, followed by intensity normalization via Z-score standardization computed from the foreground voxels within the segmented thyroid nodule region, with robust percentile clipping (1st–99th percentiles) applied prior to standardization to suppress outlier effects. For gray-level discretization during texture feature extraction, a fixed bin width of 25 HU-equivalent intensity units was applied, which was determined from the normalized intensity range of the reference tissue rather than from the global image, in line with IBSI recommendations.

All thyroid nodules were manually segmented by two ultrasonographers (each with ≥5 years of experience) using ITK-SNAP (v3.8.0), with segmentations saved as NII files. Intra- and inter-observer reproducibility were assessed using the intraclass correlation coefficient (ICC) based on 100 randomly selected images exclusively sampled from the training set, with all observers blinded to pathological findings and Ki-67 status. Intra-observer reproducibility was evaluated by having the same observer repeat segmentation after a two-week interval, while inter-observer reproducibility was assessed by having two observers independently segment the same image set. This process ensured feature stability and minimized noise in subsequent modeling. Based on the preprocessed images, a total of 1,070 radiomics features were extracted using IFoundry software. These comprised first-order statistical intensity features (n, 18); texture features, including gray-level co-occurrence matrix (GLCM, n, 15), gray-level run-length matrix (GLRLM, n, 12), gray-level size zone matrix (GLSZM, n, 21), neighboring gray-tone difference matrix (NGTDM, n, 18), and gray-level dependence matrix (GLDM, n, 17); as well as contour features (n, 5), shape features (n, 13), and textural phenotype features (n, 13). In addition, high-order features were derived from wavelet (n, 432), Gabor (n, 468), and local ternary pattern (LTP) filters (n, 48).

Feature screening was performed using a sequential approach. First, all features were normalized using Min-Max scaling to the [0, 1] range. Features with an intraclass correlation coefficient (ICC) ≥ 0.8 were retained to ensure reproducibility. Candidate features were then identified via univariate analysis using either the independent samples t-test or Mann-Whitney U test (*P* < 0.05). Redundant features with a Spearman correlation coefficient > 0.9 were removed. Finally, the optimal feature subset was selected using least absolute shrinkage and selection operator (LASSO) regression to mitigate overfitting. All feature engineering steps were carried out only within the training set. All feature engineering steps, including reference-tissue-based normalization and fixed-bin-width discretization, were performed exclusively within the training set using parameters derived from training-set reference regions; the corresponding parameters were then stored and applied identically to the validation and testing sets without re-estimation, strictly following IBSI guidelines to prevent data leakage and ensure methodological rigor.

### 2.5 Model development and validation

Two basic models were initially developed to establish a performance baseline. The first was a conventional ultrasound (US) model, which incorporated visual US features that were significantly associated with high Ki-67 expression. The second was a radiomics model (Rad), constructed using optimally selected quantitative radiomics features. To evaluate the potential benefit of multimodal integration, two combined models were subsequently constructed. The Rad+US model integrated both radiomics features and visual US predictors, while the Rad+US+Gene model further incorporated BRAF V600E and TERT promoter mutation status.

To address the class imbalance (94/627, 15.0% positive), the random forest classifier was employed with class_weight=“balanced”, assigning inverse class-frequency weights to penalize minority-class misclassifications. For model construction, we performed systematic hyperparameter tuning using five-fold cross-validation within the training set. The searching ranges were set as follows: n_estimators (50, 100, 150, 200, 250, 300), max_depth (3–5, 6, 8, None), min_samples_split (2, 3, 5, 8), and split criterion (gini, entropy), and class_weight was fixed at “balanced” during tuning to maintain consistent imbalance correction. The final model was selected according to the highest cross-validation AUC. The optimal hyperparameters were n_estimators, 200, max_depth, 5, min_samples_split, 3, criterion, gini, class_weight=“balanced”, and random_state=42.

### 2.6 Statistical analysis

Continuous variables were expressed as mean ± standard deviation (SD) if normally distributed, or as median with interquartile range otherwise. Categorical variables were presented as counts and percentages, with between-group comparisons performed using the chi-square test or Fisher’s exact test, as appropriate. Radiomics feature screening was conducted using a sequential approach as detailed above.

For model development, predictive performance was evaluated using receiver operating characteristic (ROC) analysis, with AUC and its 95% confidence interval (CI) reported. Model discrimination capability was classified as good (AUC > 0.7) or excellent (AUC > 0.8). Additionally, the Brier score was used to assess the calibration of the model (**Supplementary Table 2**), and decision curve analysis (DCA) was performed to evaluate the clinical utility of prediction models by quantifying the net benefit at different threshold probabilities. Sensitivity, specificity, PPV, NPV, Recall, F1 score, Balanced Acc, MCC and accuracy odds ratio (OR) were also calculated. Pairwise AUC comparisons were performed using the DeLong test with Benjamini–Hochberg FDR correction, to assess whether multimodal integration improved predictive performance for high Ki-67 expression in PTC. Stratified sampling was employed to validate model stability. All statistical tests were two-sided, and a *P* value < 0.05 was considered statistically significant. Analyses were conducted using SPSS (version 25.0) and Python (version 3.13).

## 3 Results

### 3.1 Clinical characteristics of patients

This study included 627 patients with papillary thyroid carcinoma (PTC) who underwent immunohistochemical assessment of Ki-67 proliferation index, of whom 94 (15.0%) had high expression (Ki-67 ≥ 5%) and other 533 had low expression (Ki-67 < 5%). The mean age was 45.1 ± 13.8 years. High Ki-67 expression was significantly associated with BRAF V600E mutation, TERT promoter mutations, and dual mutations (all *P* < 0.05). All gene mutation tests were performed using preoperative FNA samples, with 611 cases further validated via postoperative surgical specimens. Regarding clinicopathological features, significant differences between the high and low Ki-67 expression groups were observed in age, lymph node metastasis, and advanced TNM stage (all *P* < 0.05), whereas no significant difference was found in sex (*P* > 0.05). (**Table 1**).

**Table 1:** Comparison of clinical and pathological baseline characteristics of PTC patients with different Ki-67 expression groups.

Features	Aggregate (n=627)	Ki-67 ≥ 5% (n=94)	Ki-67<5% (n=533)	*P*
*BRAF V600E* mutation, n (%)	511 (81.4)	84 (89.3)	427 (80.1)	0.033*
*TERT*-p mutations, n (%)	30 (4.7)	17 (18.0)	13 (2.4)	0.000*
Dual mutations, n (%)	28 (4.4)	16 (17.0)	12(2.2)	0.000*
Age, years ^a^	45.1 ± 13.8 (18–87)	49.3 ± 15.0 (18-84)	44.3 ± 13.4 (18-87)	0.012*
Male, n (%)	186 (29.6)	33 (35.1)	153 (28.7)	0.222
Lymph node metastasis, n (%)				0.010*
No	289 (46.0)	27 (28.7)	262 (49.1)	
Central	261 (41.6)	45 (47.8)	216 (40.5)	
Lateral	77 (12.2)	22 (23.4)	55 (10.3)	
TNM III+IV, n (%)	185 (29.5)	49 (52.1)	136 (25.5)	0.000*

^a^ Mean ± standard deviation (range).

* Statistically significant difference.

### 3.2 Univariate analysis: correlation between visual US features and Ki-67 expression

**Figure 2:**
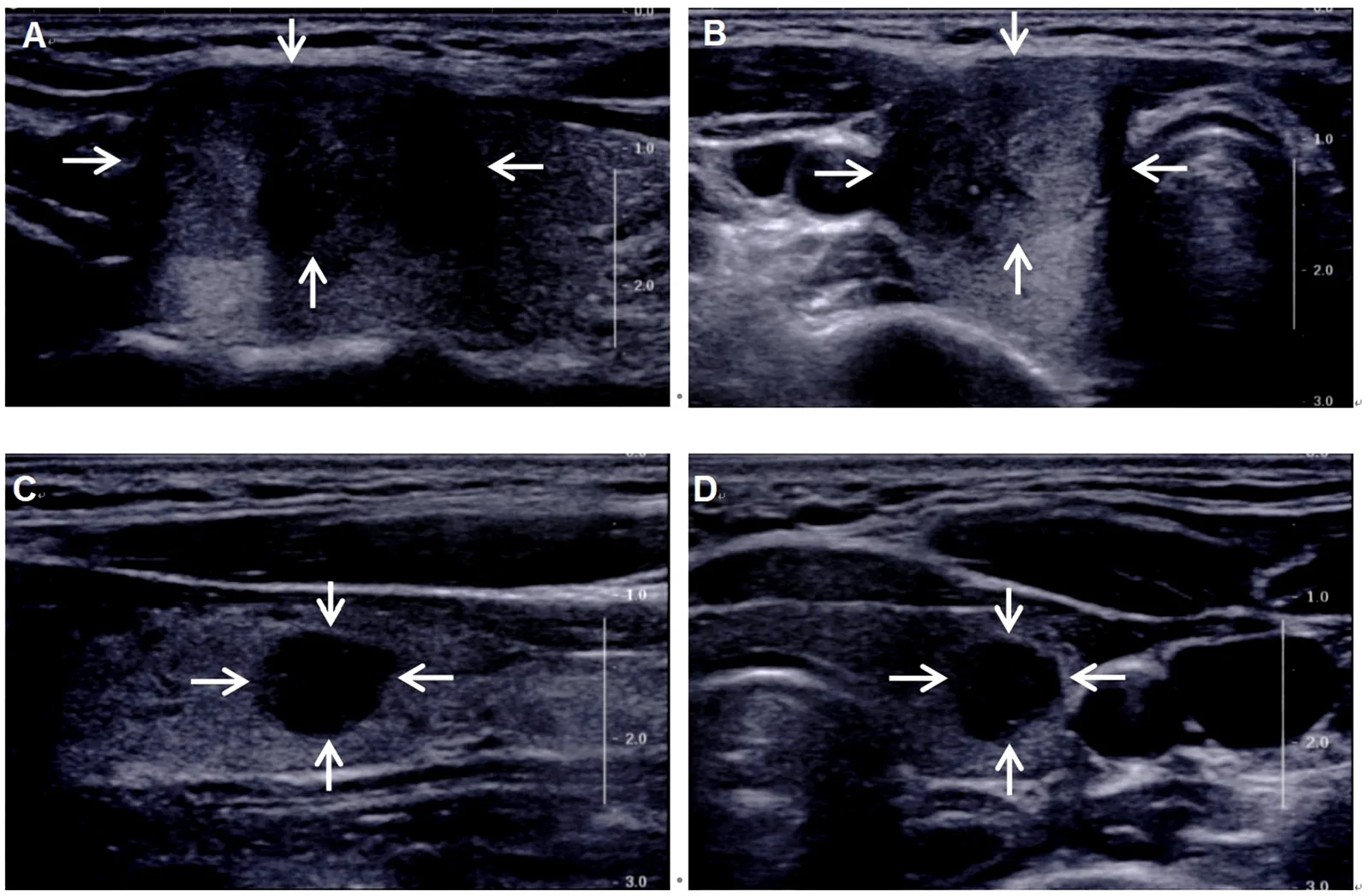
Ultrasound images comparing high versus low Ki-67 expression. **(A, B)**: images from a patient with Ki-67 expression ≥ 5% (Case 1); **(C, D)**: images from a patient with Ki-67 expression < 5% (Case 2). The lesion is indicated by white arrows.

Among the 627 patients with PTC, the median tumor size was 17.0 ± 7.0 mm in the low Ki-67 expression group and 21.8 ± 16.8 mm in the high expression group. Multifocality was observed in 124 patients with low expression and 57 with high expression. To further evaluate the association between ultrasound features and Ki-67 expression, univariate analysis was performed. Ki-67 ≥ 5% was significantly associated with maximum tumor diameter, capsular invasion, and multifocality (all *P* < 0.001), whereas no significant differences were identified for nodule location, taller-than-wide shape, calcification, marked hypoechogenicity, posterior acoustic features, blood flow signal, or vascular distribution (all *P* > 0.05). Detailed results are presented in **Table 2** and US images illustrating high and low Ki-67 expression are presented in **Figure 2**.

**Table 2:** Univariate Analysis of the correlation between visually US features and Ki-67 expression level.

US Features	Ki-67 ≥ 5% (n=94)	Ki-67 < 5% (n=533)	*P*
**Upper/Middle location, n** (**%**)	57 (60.6)	284 (53.4)	0.187
**Multifocality, n** (**%**)	70 (74.4)	124 (33.2)	0.000*
**Maximum dia meter**, **mm**	21.8 ± 16.8	17.0 ± 7.0	0.000*
**Taller-than-wide**, **n( %**)	50 (53.2)	247 (46.3)	0.263
**Capsule invasion**, **n (%))**	71 (75.5)	250 (46.9)	0.000*
**Calcification**, **n** (**%**)			0.130
No	33 (35.1)	244 (45.8)	
Microcalcification	50 (53.2)	227 (42.6)	0.290
Macrocalcification	11 (20.3)	62 (11.6)	0.739
**Solid composition, n (%))**	87 (92.6)	497 (93.2)	0.825
**Markedly hypoechoic**, **n** (**%**)	32 (34.0)	162 (30.4)	0.545
**Posterior features, n** (**%**)			0.157
No	48 (51.1)	328 (61.5)	
Enhancement	29 (25.8)	133 (25.0)	0.502
Shadowing	17 (18.1)	72 (13.5)	0.344
**Rich blood flow signal**, **n** (**%**)	12 (12.8)	70 (13.1)	0.157
**Vascularity, n** (%)			0.293
Peripheral	68 (72.3)	415 (77.9)	
Centra	2 (2.2)	17 (3.2)	0.696
Mixed	24 (25.5)	101 (18.9)	0.102

* Statistically significant difference. Bold text indicates the US features that were included in the final analysis.

### 3.3 Factor screening and determination of high Ki-67 expression prediction model

To identify independent predictors associated with high Ki-67 expression (≥5%) in PTC, multivariate logistic regression analysis was performed on variables that showed significant differences (*P* < 0.05) in baseline clinicopathological and univariate analyses. After multivariate analysis, age, maximum tumor diameter, multifocality, and capsular invasion were identified as independent predictors of high Ki-67 expression (all *P* < 0.05). The corresponding ORs were as follows: age (OR, 1.020, 95% CI: 1.002–1.039, *P*, 0.034); maximum tumor diameter (OR, 1.044, 95% CI: 1.016–1.073, *P*, 0.002); multifocality (OR, 8.919, 95% CI: 5.244–15.170, *P* < 0.001); and capsular invasion (OR, 3.625, 95% CI: 2.067–6.358, *P* < 0.001). (**Table 3**).

**Table 3:** Multivariate logistic regression analysis of predictive factors for Ki-67 high expression based on visually ultrasound features.

Features	B	SD	OR (95% CI)	*P*
Age	0.020	0.009	1.020 (1.002-1.039)	0.034*
Maximum diameter	0.043	0.014	1.044 (1.016-1.073)	0.002*
Multifocality	2.188	0.271	8.919 (5.244-15.170)	0.000*
Capsule invasion	1.288	0.287	3.625 (2.067-6.358)	0.000*

B, Regression coefficient, SE, Standard error, OR= Odds ratios.

* Statistically significant difference

### 3.4 Radiomics features reduction and screening

After applying the predefined dimensionality reduction and screening criteria, 661 ICC ≥ 0.8 radiomics features with good reproducibility (**Supplementary Table 3**) and 160 Spearman rank correlation coefficient ≤ 0.9 were retained. Univariate analysis further narrowed these to 133 features significantly associated with Ki-67 expression. Following LASSO regression, eight features with non-zero coefficients were identified as key predictors of high Ki-67 expression. Detailed information on these 8 key radiomics features is summarized in **Supplementary Table 4**.

### 3.5 Model development and validation

**Figure 3:**
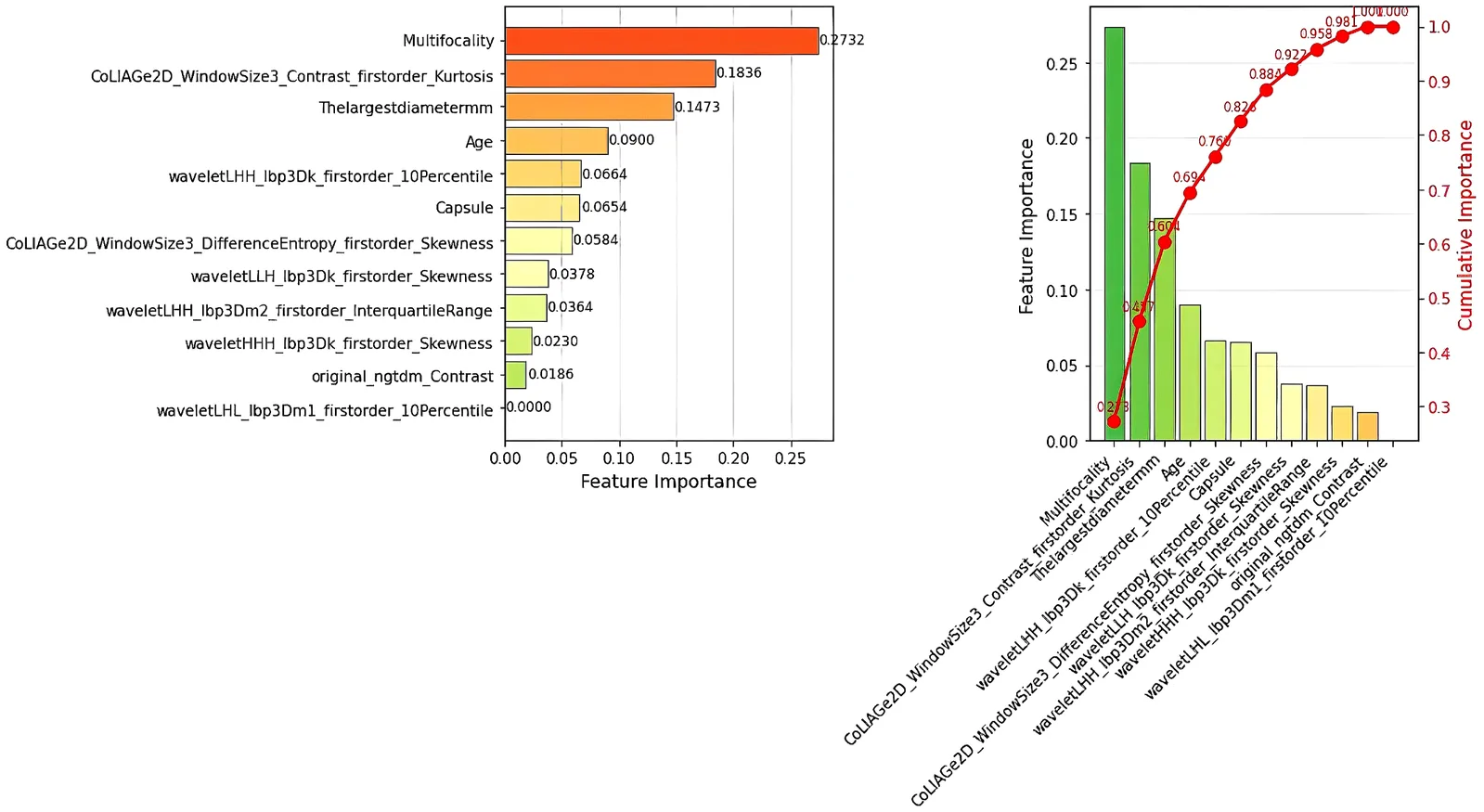
Predict the ranking of the importance of the high Ki-67 expression.

**Figure 4:**
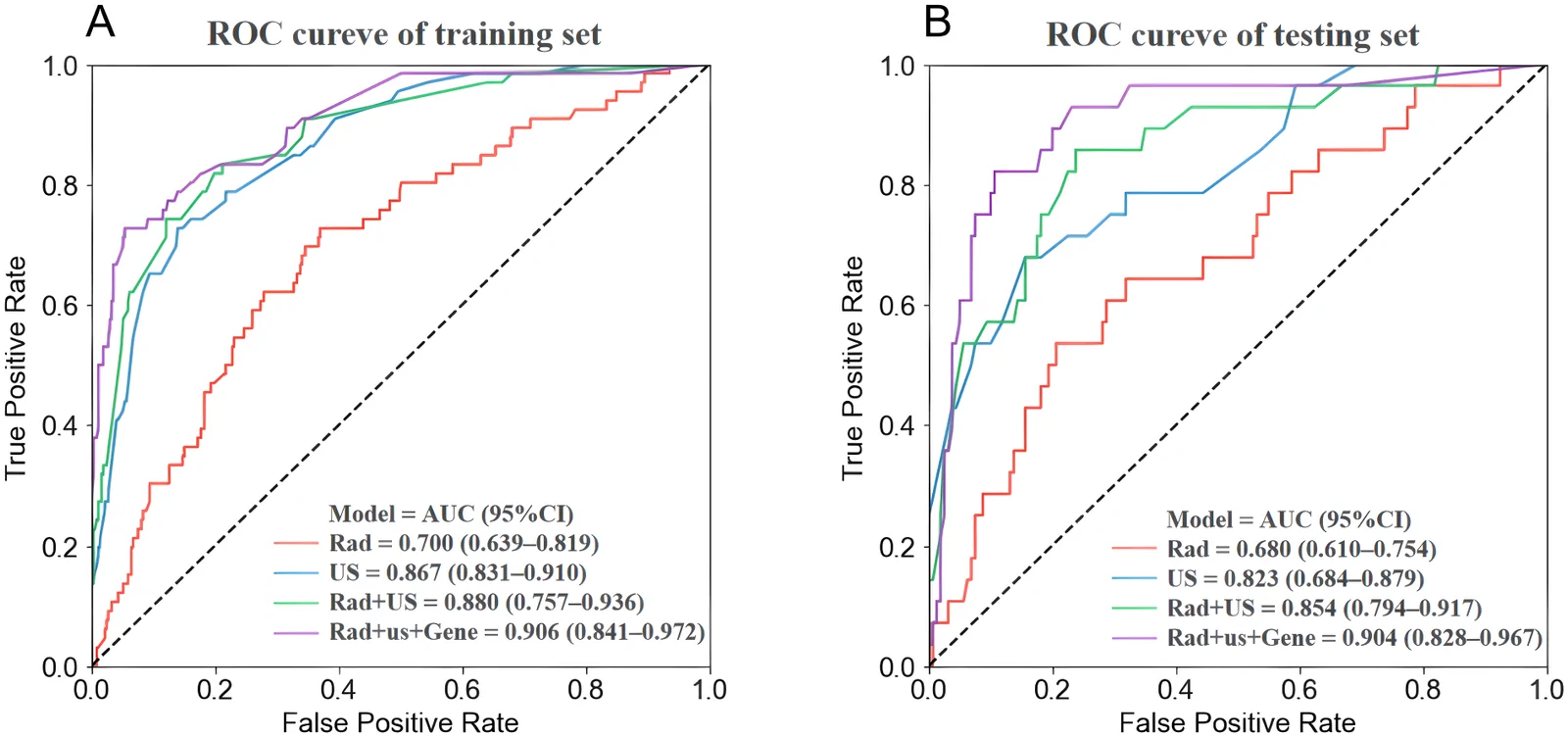
ROC curves of Ki-67 prediction using different models.

Four predictive models were constructed, including US and Rad baseline models and Rad+US and Rad+US+Gene combined models. **Figure 3** corresponds to the integrated Rad+US+Gene model with all risk predictor factors. The Rad model, built on the eight selected radiomics features, achieved an AUC of 0.700 (95% CI: 0.639–0.819) in the training set and 0.680 (95% CI: 0.610–0.754) in the testing set. In contrast, the US model built with the four significant ultrasound features exhibited superior performance, with AUCs of 0.867 (95% CI: 0.831–0.910) and 0.823 (95% CI: 0.684–0.879) in the training and testing sets, respectively. The combined Rad+US model further enhanced predictive accuracy, yielding AUCs of 0.880 (95% CI: 0.757–0.936) in the training set and 0.854 (95% CI: 0.794–0.917) in the testing set. Notably, the tri-modal Rad+US+Gene model, which additionally incorporated BRAF V600E and TERT promoter mutations, achieved the optimal predictive performance, with the highest AUCs of 0.904 (95% CI: 0.841–0.972) in the training set and 0.906 (95% CI: 0.828–0.967) in the testing set.(**Table 4**, **Figure 4**).

**Table 4:** Diagnostic performance of four methods for predicting high expression of Ki-67.

Evaluation index	Training set (n=439)	Testing set (n=188)
Rad model		
AUC (95% CI)	0.700 (0.639–0.819)	0.680 (0.610–0.754)
Accuracy, %	85.2 (79.2–90.1)	85.1 (79.7–90.0)
Specificity, %	71.0 (61.4–79.6)	62.5 (52.4–72.0)
Sensitivity, %	80.3 (71.7–87.2)	78.6 (69.4–85.8)
US model		
AUC (95% CI)	0.867 (0.831–0.910)	0.823 (0.684–0.879)
Accuracy, %	85.7 (80.3–89.9)	85.1 (79.6–89.4)
Specificity, %	83.1 (75.5–88.7)	80.6 (72.1–86.8)
Sensitivity, %	74.2 (66.1–83.9)	75.0 (66.9–84.6)
Rad+US model		
AUC (95% CI)	0.880 (0.757–0.936)	0.854 (0.794–0.917)
Accuracy, %	86.1 (79.3–91.1)	86.2 (79.4–91.2)
Specificity, %	81.8 (72.5–88.8)	82.9 (73.6–89.7)
Sensitivity, %	81.8 (70.4–89.3)	77.7 (67.9–85.4)
Rad+US+Gene model		
AUC (95% CI)	0.906 (0.841–0.972)	0.904 (0.828–0.967)
Accuracy, %	90.0 (83.4–94.6)	88.3 (81.2–93.3)
Specificity, %	84.4 (74.9–91.2)	85.7 (76.4–92.1)
Sensitivity, %	89.3 (80.2–95.0)	85.6 (76.1–92.1)

Rad, Radiomics; US, Ultrasound; Gene =BRAF V600E and TERTpomoter.

### 3.6 Model performance and comparison

**Figure 5:**
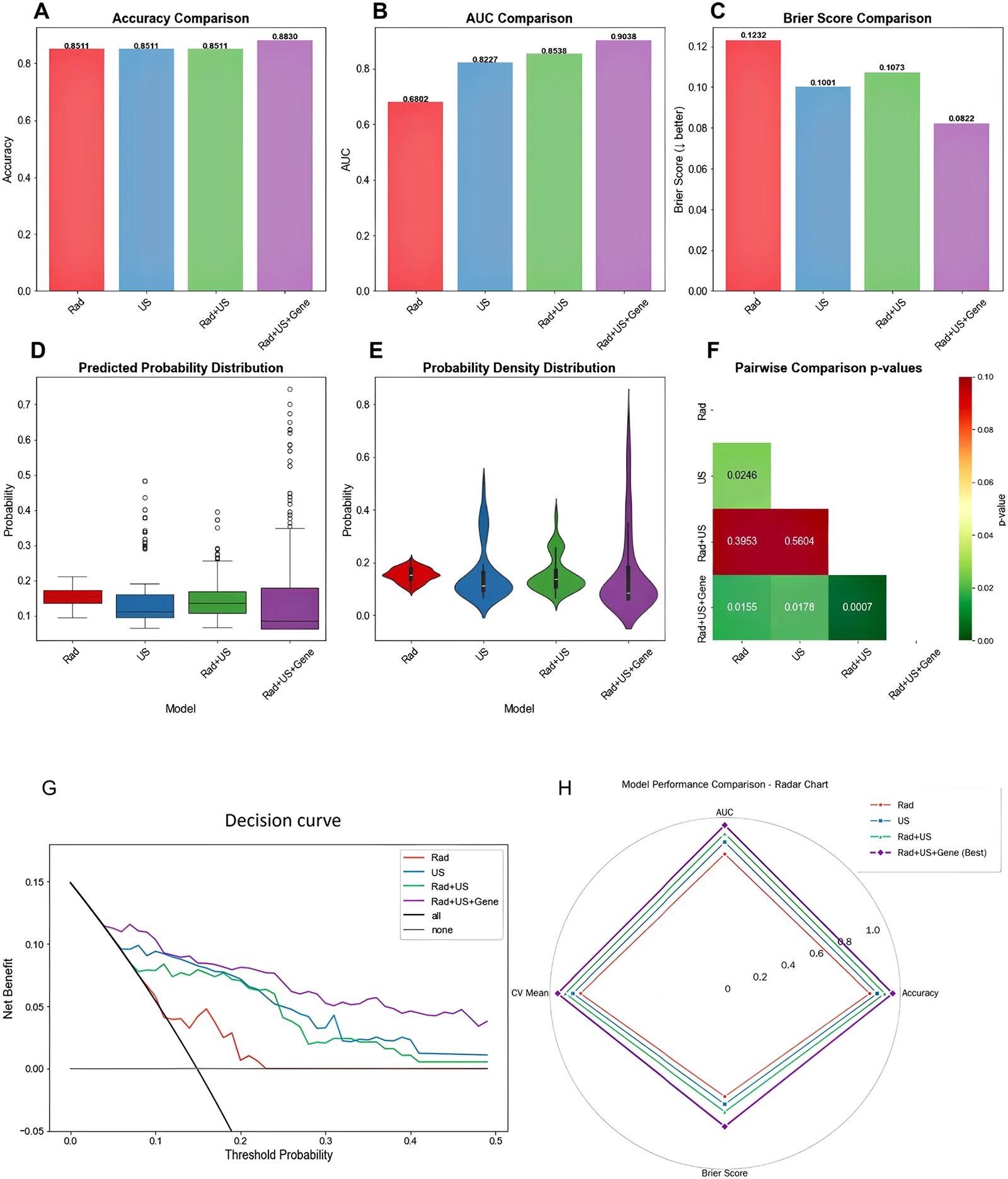
Comparative analysis of Ki-67 predictions by different models. **(A)** Comparison chart of prediction accuracy; **(B)** AUC bar comparison chart; **(C)** Brier Score; **(D)** Box plot **(E)** for predictive performance: violin plot for predictive performance; **(F)** Compare the P-value matrix heatmap of pairwise models; **(G)** Comparison of multiple model decision curves; **(H)** Comprehensive comparison chart of performance of various models.

To evaluate the incremental value, we compared the predictive performances across four models: Rad, US, Rad+US, and Rad+US+Gene. Accuracy, PPV, NPV, Recall, F1 score, Balanced Acc and MCC were all highest in the Rad+US+Gene model, followed by the Rad+US model, while the baseline Rad model and the US model generally performed less favorably (**Figure 5A**, **Supplementary Table 5**). A progressive increase in AUC was observed from Rad (0.680) to US (0.823), Rad+US (0.854), and Rad+US+Gene (0.904) models (**Figure 5B**). The Rad+US+Gene model also achieved the lowest Brier score (0.0822), compared with 0.1232 for the Rad model, indicating superior calibration (**Figure 5C**, **Supplementary Figure 1**). Probability density analysis further revealed that this model generated high-confidence predictions and provided the strongest risk stratification (**Figures 5D, E**).

Consistent with our pre-specified analytic plan that designated the AUC as the primary endpoint, the Rad+US+Gene model significantly outperformed the Rad (adjust *P*, 0.011), US (adjust *P*, 0.032), and Rad+US (adjust *P*, 0.001) models (**Figure 5F**, **Table 5**). Decision curve analysis confirmed that this model provided the highest net benefit across a threshold probability range of 0.1–0.5 (**Figure 5G**). Collectively, the integrated Rad+US+Gene model demonstrated superior performance across all evaluated metrics, including accuracy, AUC, and Brier score, establishing it as the most reliable predictive model for Ki-67 expression status in this study (**Figure 5H**).

**Table 5:** Comparison of four methods for predicting high ki-67 expression.

Models	Accuracy	AUC	ΔAUC (vs Rad+US+Gene)	C V mean	Brier score	Adjusted *P* (vs Rad+US+Gene)
Rad	0.8511	0.6802	0.2236	0.8515	0.1232	0.011*
US	0.8513	0.8227	0.0811	0.8561	0.1001	0.032*
Rad+US	0.8617	0.8538	0.0500	0.8567	0.1073	0.001*
Rad+US+Gene	0.8830	0.9038	—	0.8781	0.0822	

CV mean, Cross validation mean.

* Statistically significant difference

## 4 Discussion

Preoperative assessment of Ki-67 expression status has important clinical significance due to its prognosis value (28, 29) and the pervasive intratumoral heterogeneity that affects the reliability of FNA (30). As a classic proliferation marker, the expression level of Ki 67 is closely related to the biological behavior, invasion potential, and clinical outcomes of tumors (13–31). This study systematically constructed and evaluated four predictive models of radiomics (Rad), ultrasound (US), radiomics combined with ultrasound (Rad+US), and radiomics+ultrasound+gene multimodal integration (Rad+US+Gene) prediction models for preoperative evaluation of Ki-67 expression status. The results showed that the integrated model combined radiomics, ultrasound, and gene features exhibited superior overall performance in accuracy, AUC and Brier score, confirming the value of multimodal feature fusion in improving the predictive efficiency of High ki-67 expression.

Ultrasound radiomics, as an emerging technology that combines medical imaging and bioinformatics, breaks the limitations of traditional ultrasound that can only provide qualitative descriptions. It can extract a large number of quantitative features from ultrasound images that cannot be recognized by the naked eye, including morphology, grayscale histogram, texture, and higher-order features (32). These features can objectively reflect intratumoral heterogeneity, which serves as a vital imaging biomarker characterizing biological behaviors of tumors including proliferative activity and invasive potential (33, 34). In recent years, radiomics has been extensively employed in the diagnosis (35, 36), staging (37), prognostic evaluation (38), and gene mutations prediction (39) of PTC, exhibiting promising clinical application prospects. This provides a feasible non-invasive method for predicting Ki-67 expression. This study revealed that the incorporated radiomics features, including entropy, contrast, kurtosis, skewness, percentiles, and interquartile range, collectively demonstrated that greater heterogeneity in the intratumoral grayscale distribution and more intricate textural architecture correspond to elevated tumor heterogeneity. Increased tumor heterogeneity is generally tightly associated with vigorous tumor cell proliferation and heightened invasive potential. These findings align with the biological implication of high Ki-67 expression, a biomarker indicative of robust cellular proliferative activity. To the best of our knowledge, this study is the first to explore the association between ultrasound radiomics signatures and Ki-67 overexpression in PTC.

Previous studies have found a correlation between conventional ultrasound features and Ki 67 expression in medullary thyroid cancer (40). Nevertheless, ultrasound signatures linked to elevated Ki-67 expression in PTC have yet to be characterized. In the present study, we found that larger tumor size, capsular invasion, and multifocality on conventional ultrasound were significantly associated with high Ki-67 expression. Additionally, eight radiomics features derived from ultrasound images exhibited a slight correlation with high Ki-67 expression. These findings not only validate the prior conclusions of previous research but also address the existing research gap by explicitly identifying the ultrasound features linked to high Ki-67 expression in PTC.

The predictive performance of the basic prediction model constructed using the selected radiomics features was moderate, with an area under the curve (AUC) of 0.680, whereas the basic model built based on ultrasound features achieved an AUC of 0.823. Notably, the combination of these two models yielded a higher AUC of 0.854. This integration significantly enhances the clinical utility of ultrasound, providing a non-invasive, cost-effective approach for the preoperative prediction of Ki-67 expression in PTC. Ultimately, this method may facilitate more accurate risk stratification and personalized clinical management of patients with PTC.

Furthermore, Ki-67 expression is not isolated but associated with PTC-related gene mutations. BRAF V600E and TERT promoter double mutations, well-recognized in aggressive PTC, activate the MAPK signaling pathway and upregulate Ki-67 expression (41). We incorporated integrated these mutational statuses with radiomic and ultrasound features into a combined model (Rad+US+Gene). This model achieved optimal discrimination and calibration, as reflected by AUC, accuracy, PPV, NPV, Recall, F1 score, Balanced Acc, MCC and Brier score, consistent with prior studies demonstrating the advantages of multi-omics fusion (42). Pairwise comparisons showed statistically significant differences between the combined model and the Rad, US, and Rad+US models, demonstrating the complementary value of imaging phenotypes and genomic data. Mutation status alone, however, is insufficient for predicting Ki-67 expression. No clinical guideline currently recommends BRAF/TERT mutation status as a surrogate biomarker for Ki-67 expression.Ki-67 immunohistochemistry is a postoperative risk-stratification index, while preoperative molecular testing primarily guides surgical decision-making rather than estimating proliferative phenotype. Substantial genotype–phenotype heterogeneity exists: co-mutated micro-PTCs may exhibit low Ki-67 despite aggressive genotypes, and wild-type lesions can display high proliferative activity. Additionally, genetic analysis provides only static binary information, whereas ultrasound and radiomics capture global tumor phenotypes. These constraints motivate multimodal clinical evaluation instead of isolated genetic interpretation. These findings provide objective, stable predictive factors for preoperative quantitative evaluation of Ki-67 expression, and a reference for further assessing tumor biological behavior and guiding personalized treatment.

The multimodal Rad+US model works well as a first-line triage tool in community hospitals without molecular facilities. When patients are transferred to tertiary centers for definitive management, adding gene information further improves risk stratification, especially for borderline ultrasound cases. To further illustrate the tiered diagnostic pathway and enhance translational value, we propose a stepwise clinical workflow combined conventional ultrasound, radiomics triage, selective molecular testing and clinical decision-making (**Supplementary Figure 2**).

Despite these promising findings, several limitations should be addressed in future research. First, as a single-center retrospective study with a relatively small sample, selection bias was unavoidable, which is inherent to this study design. Second, manual ROI segmentation was applied. Although double-blind annotation and ICC analysis were used to minimize subjective bias, the influence of human factors could not be fully eliminated. Third, only common mutations including BRAF V600E and TERT promoter were analyzed, while other genetic variants potentially regulating Ki-67 expression were not included, which may lead to the loss of biologically meaningful information. Fourth, this study solely utilized Random Forest model. Despite its strengths in handling high-dimensional data, nonlinear feature interactions and moderately sized imbalanced datasets, we did not benchmark it against mainstream classifiers including Logistic Regression, XGBoost and SVM, which merits further exploration. Finally, the generalization capability of the model remains to be validated. Model performance was only assessed via internal validation using training and test datasets, without external validation from multi-center and large-scale cohorts.

## 5 Conclusion

This study identified an association between ultrasound radiomics signatures and Ki-67 expression in PTC. Optimized radiomics features, after rigorous screening, showed moderate ability to stratify PTC patients into high and low Ki-67 subgroups. We also developed a prediction model combining these radiomics features with visual ultrasound findings, enabling noninvasive preoperative assessment of Ki-67 with favorable efficacy. Furthermore, Ki-67 expression correlated closely with BRAF V600E, TERT promoter mutations, and their co-mutations, with a significantly higher high Ki-67 expression in mutation-carrying than wild-type groups. Importantly, integrating genetic mutations profiles with radiomics and US features substantially improved model performance in predicting high Ki-67 expression. It may help stratify patients for more aggressive surgical strategies versus conservative management. The combined model exhibited superior accuracy and clinical applicability, supporting its potential as a candidate method for preoperative risk stratification. Nevertheless, these findings should be considered preliminary because of no external validation, and further multi-center cohorts are required to confirm the generalizability of our results.

## Data Availability

The original contributions presented in the study are included in the article/**Supplementary Material**. Further inquiries can be directed to the corresponding authors.
