# Supplementary material for: Ultrasound radiomics for preoperative evaluation of Ki-67 proliferation index in papillary thyroid carcinoma
Source: Front Oncol. 2026 Sep 3;16:1881562. doi: 10.3389/fonc.2026.1881562 (PMC13581947; doi:10.3389/fonc.2026.1881562)
Supplement: Supplementary file 1 [file Table1.docx]

***Supplementary Material***

# 1 Supplementary Tables

**Table S1 Information of machine used to collect images data**

| Machine | Total number | Proportion | Transducer frequency (MHz) |
| --- | --- | --- | --- |
| Supersonic Imagine | 481 | 76.7% | 5-14 |
| GE Healthcare Logiq E9 | 146 | 23.3% | 6-15 |

**Table S2 Brier score interpretation**

| Brier Score | Interpretation | Description |
| --- | --- | --- |
| 0.000 | Perfect | Predictions are exactly correct (prob = 1 when event occurs, 0 when it does not) |
| 0.01–0.05 | Excellent | Predictions are highly accurate; very close to observed outcomes |
| 0.06–0.10 | Very good | Strong predictive accuracy; well-calibrated probabilities |
| 0.11–0.20 | Good | Reliable predictions; moderate error |
| 0.21–0.25 | Fair/Marginal | Slightly better than random guessing |
| 0.25 (baseline) | Random guess | Equivalent to always predicting 0.5 (binary) |
| 0.26–0.35 | Poor | Predictions are weak; notable error |
| 0.36–0.50 | Very poor | High error; poorly calibrated |
| > 0.50 | Extremely poor | Predictions are often wrong or strongly biased |

**Table S3 Descriptive Statistics of Intra-Class Correlation Coefficients (ICC)**

| Statistic | Intra_ICC | Inter_ICC |
| --- | --- | --- |
| Mean | 0.9708 | 0.9623 |
| Median | 0.9840 | 0.9815 |
| Standard Deviation | 0.0339 | 0.0444 |
| Minimum | 0.8079 | 0.8004 |
| Maximum | 1.0000 | 1.0000 |
| 25th Percentile | 0.9625 | 0.9431 |
| 75th Percentile | 0.9913 | 0.9946 |

**Table S4 Significant radiomics features associated with high Ki‑67 expression**

| Radimoic features | Importance Score | Biological Interpretation |
| --- | --- | --- |
| Original ngtdm Contrast | 0.0186 | Reflects local gray-level differences; higher values indicate more pronounced intensity variations between adjacent voxels within the tumor. |
| CoLIAGe2D WindowSize3 Contrast firstorder Kurtosis | 0.1836 | Reflects the peakedness of the contrast image distribution; high kurtosis suggests gray-level variations concentrated within a narrow range, indicating relatively homogeneous tissue composition. |
| CoLIAGe2D WindowSize3 Difference.Entropy firstorder Skewness | 0.0584 | Reflects the asymmetry of the difference entropy image distribution; positive skewness indicates dominance of high-value regions, negative skewness indicates dominance of low-value regions. |
| Wavelet.LLH lbp.3D.k firstorder Skewness | 0.0378 | Reflects the skewness of the LBP texture map in the LLH subband, characterizing the asymmetry of high-frequency detail textures. |
| Wavelet.LHL lbp.3D.m1 firstorder 10Percentile | 0.0000 | Reflects the intensity level of low-gray regions; smaller values suggest more extensive micro-necrosis, micro-cystic changes, or loose architectural areas. |
| Wavelet.LHH lbp.3D.m2 firstorder InterquartileRange | 0.0364 | Reflects the dispersion of gray-level distribution at lesion margins and in 3D micro-texture maps; higher values indicate more uneven grayscale distribution. |
| Wavelet.LHH lbp.3D.k firstorder 10Percentile | 0.0664 | Quantifies the proportion of weak-gray low-texture regions; lower values suggest more necrosis/uniform areas, higher values indicate greater heterogeneity and complex texture. |
| wavelet.HHH_Ibp.3D.k_firstorder_Skewness | 0.0230 | Reflects skewness of the LBP texture map derived from the HHH wavelet subband, characterizing asymmetry in high‑frequency isotropic textural details across all three dimensions. |

**Table S5 Diagnostic performance for predicting of high Ki-67 expression**

| **Model** | **PPV**  **(95% CI)** | **NPV**  **(95% CI)** | **Recall** | **F1 score** | **Balanced Acc** | **MCC** |
| --- | --- | --- | --- | --- | --- | --- |
| **Training set** | | | | | | |
| Rad-model | 0.3288  (0.2451–0.4227) | 0.9532  (0.9142–0.9765) | 0.7030 | 0.4666 | 0.7565 | 0.4804 |
| US-model | 0.4372  (0.3354–0.5479) | 0.9479  (0.8975–0.9748) | 0.7420 | 0.5502 | 0.7865 | 0.5698 |
| Rad+US | 0.4430  (0.3444–0.5518) | 0.9621  (0.9284–0.9811) | 0.8180 | 0.5747 | 0.8180 | 0.6076 |
| Rad+US+Gene | 0.5032  (0.3731–0.6483) | 0.9781  (0.9498–0.9910) | 0.8930 | 0.6437 | 0.8685 | 0.6956 |
| **Testing set** | | | | | | |
| Rad-model | 0.2684  (0.1673–0.3986) | 0.9435  (0.8703–0.9780) | 0.7860 | 0.4001 | 0.7055 | 0.3951 |
| US-model | 0.4035  (0.2642–0.5617) | 0.9485  (0.8854–0.9792) | 0.7500 | 0.5247 | 0.7780 | 0.5424 |
| Rad+US | 0.4430  (0.2946–0.6043) | 0.9550  (0.8941–0.9832) | 0.7770 | 0.5642 | 0.8030 | 0.5911 |
| Rad+US+Gene | 0.5116  (0.3543–0.6674) | 0.9714  (0.9187–0.9912) | 0.8560 | 0.6404 | 0.8565 | 0.6869 |

# 2 Supplementary Figures

**Figure S1** Calibration curves of four predictive models

**Figure S2** Multimodal workflow combining conventional ultrasound, radiomics, and molecular testing for preoperative evaluation of Ki-67 expression in PTC
